# Management of multiple pregnancy with an affected twin

**DOI:** 10.1186/1824-7288-41-S1-A17

**Published:** 2015-09-24

**Authors:** Mario Giuffrè, Davide Vecchio, Simona La Placa, Giuseppa Pinello, Ettore Piro, Ingrid Anne MandySchierz, Giovanni Corsello

**Affiliations:** 1 Dipartimento di Scienze per la Promozione della Salute e Materno Infantile “G. D'Alessandro”, Università degli Studi di Palermo, Via A. Giordano 3, 90127 Palermo, Italy

## 

Newborns from multiple pregnancies demonstrate a higher perinatal morbidity and mortality compared to singletons. Prematurity is more frequent in twins and therefore birth weight is significantly lower compared to singletons [1]. Thus, twins are more exposed to prematurity related diseases (respiratory, cardiovascular, infectious, etc.) and to long-term complications [2]. It is very difficult to estimate the increased risk of neonatal morbidity related to twinning independently to the increased risk of prematurity. Prematurity is the main reason for most neonatal diseases in twins, but other variables may play a role. Fetal growth restriction [3] and congenital malformationsare major issues in offspring of multiple pregnancies. Specific risks vary according tozigosity (monozygotic >dizygotic) and kind (genetic, vascular, multifactorial, etc.) and site (systems and organs involved) of malformation. Accurate risk assessment strategies and adequate obstetrical-neonatological management of multiple pregnancies may reduce the increasing need for neonatal intensive care and for health resources in the long-term follow-up that has been observed over the last decades.

Careful analysis of both twins for a pathological condition is mandatory to address the most appropriate management. Twin discordance for the presence of a severe pathological condition raises serious concern in terms of bioethical and psychological impact on the parents and medical staff[4]. Different management choices can be considered: termination of pregnancy, selective embryo reduction of the affected twin, anticipation of delivery or natural course of the pregnancy. Each choicehides difficult clinical and legal implications. Accurate clinical, laboratory and ultrasonographic evaluation together with pregnancy follow-up are essential for reaching the correct diagnosis and consider prognosis and therapeutic options [5]. The risk of intrauterine death and potential risks for the other twin and the mother must be taken into account. Sometimes it is possible to wait until the natural end of pregnancy and then provide suitable treatment to the affected twin. Other times, parents opt to terminate the pregnancy and loose both twins. A selective reduction (after accurate evaluation of placentation) of the affected twin only carries a high risk of complication for the healthy twin, especially in monochorionic pregnancies. In the late third trimester of pregnancy, the option of a preterm delivery can be considered and may contribute to the increase of prematurity and prematurity related diseases in twins. 

The management of multiple pregnancies is a very complex task for medical staff and requires parental support with adequate counselling and psychological help [6].
